# Distance‐based assessment of spatial artifact extension in the prostate from fiducial markers in diffusion‐weighted magnetic resonance imaging

**DOI:** 10.1002/acm2.70348

**Published:** 2025-11-14

**Authors:** Mizgin Coskun, Patrik Brynolfsson, Christian Jamtheim Gustafsson, Adalsteinn Gunnlaugsson, Lars E. Olsson

**Affiliations:** ^1^ Department of Translational Medicine, Medical Radiation Physics Lund University, Carl Bertil Laurells gata 9 Malmö Sweden; ^2^ Radiation Physics, Department of Hematology, Oncology, and Radiation Physics Skåne University Hospital, Klinikgatan 5 Lund Sweden; ^3^ Division of Oncology Department of Clinical Sciences Lund Skåne University Hospital Lund University, Klinikgatan 5 Lund Sweden

**Keywords:** IM‐ MRI: Diffusion MRI, IM‐ MRI: Phantoms ‐ physical

## Abstract

**Background:**

Fiducial markers in image‐guided prostate cancer radiotherapy reduce geometric uncertainty during daily patient setup and enable assessment of target position changes. Diffusion‐weighted magnetic resonance imaging (MRI) for target delineation improves prostate cancer localization, beneficial for intraprostatic focal boost. Artifacts from fiducial markers on prostate diffusion‐weighted MRI (DWI) need to be investigated, as they could be detrimental for target delineation. This study aims to determine the distances of artifact extensions caused by fiducial markers in DWI and in the apparent diffusion coefficient (ADC) maps and to assess how motion and signal‐to‐noise ratio (SNR) influence the artifact size in ADC maps.

**Materials and methods:**

Three phantoms were used: two homogeneous gel phantoms—one containing three cylindrical gold fiducial markers (GFM) and the other containing three spherical gold anchor (GA) markers—and a third heterogeneous phantom consisting of a piece of sirloin embedded with three GFM and three GA. Diffusion‐weighted images were acquired on a 3T MRI system. The artifacts were analyzed along the phase‐encoding (PE) and frequency‐encoding (FE) directions. Motion was induced and simulated during acquisition, and SNR was varied. The impact of motion and SNR on the artifact extension was evaluated, and the artifact extensions in diffusion images from eight patients were also analyzed.

**Results:**

The artifacts were smaller in the ADC maps compared to DWI. The largest artifact extension occurred along the PE‐direction. Larger artifact extensions were observed in homogeneous phantom images compared to patient images. In homogeneous phantom images: 13.8  ±  0.4 mm / 9.1  ±  0.4 mm (PE/FE) in DWI with *b* = 0 s/mm^2^ and 11.6  ±  0.9 mm / 8.1  ±  0.4 mm (PE/FE) in the ADC map. In patient images: 10.7  ±  1.2 mm / 8.2  ±  1.3 mm (PE/FE) in DWI with *b* = 0 s/mm^2^ and 7.3  ±  1.6 mm / 6.8  ±  1.1 mm (PE/FE) in the ADC map. Motion caused larger artifact extensions compared to no motion. A motion of 2 mm increased the artifact from 11.6 ± 0.9 mm / 8.1 ± 0.4 mm (PE/FE) to 14.1 ± 0.8 mm / 9.7 ± 0.4 mm (PE/FE) in homogeneous phantom images and from 10.3 ± 0.8 mm / 8.1 ± 0.4 mm (PE/FE) to 13.1 ± 0.8 mm / 8.4 ± 0.8 mm (PE/FE) in heterogeneous phantom images. Lower SNR resulted in smaller visible artifact extensions.

**Conclusion:**

This study assessed the distances of artifact extensions in homogeneous phantoms, heterogeneous phantoms, and patient images caused by fiducial markers in DWI and ADC maps. ADC maps had smaller artifact extensions compared to DWI. The artifact extensions were largest in the homogeneous phantom, smaller in the heterogeneous phantom, and the smallest in the patient images. In patient images, the extensions were approximately 7–11 mm (PE) and 7‐8 mm (FE). However, extensions reached up to ∼14 mm (PE) and ∼9 mm (FE) in homogeneous phantom images, suggesting that the true artifact extension may be partially obscured in patient images. Further, motion in images caused larger artifact extensions, and lower SNR caused smaller artifact extensions. The study underlines the need for precise marker placement to avoid obscuring critical anatomical structures, especially for delineation of small boost volumes, and distorting ADC values in quantitative analyses of tumors.

## 1 INTRODUCTION

Since the 1990s, fiducial markers have been widely utilized for positioning in image‐guided radiotherapy for the treatment of prostate cancer.^1^, ^2^, ^3^ While the anatomical landmarks of the pelvic region can serve as a basis for daily patient setup, it does not effectively address the geometric uncertainties caused by prostate position variations. The purpose of fiducial markers is to minimize the geometric uncertainty during the daily setup of patients and variations in target position.^4^

Typically, three markers are placed into the prostate.^4^ These markers are visualized by daily volumetric or portal imaging prior to the treatment delivery. This allows for a rigid image registration between the daily image and the planning computed tomography (CT), enabling adaptation of the prostate position. With the use of three fiducial markers, the translational shift and the prostate rotation can be assessed. The current trend of hypofractionation increases the need for precise prostate position, since a higher dose will be delivered per fraction. Intraprostatic markers have become increasingly popular due to their simple use, low cost, and their ability to reduce inter‐observer variability.^4^

The visibility of fiducial markers and the image distortions they cause have been previously studied.^5^, ^6^ Ideally, the fiducial markers should be clearly visible across all imaging modalities, such as CT, cone beam CT (CBCT), magnetic resonance imaging (MRI), and planar kV‐imaging, without inducing any unwanted artifacts. A study of various fiducial markers in a pelvic phantom investigated the visibility and the presence of artifacts across multiple imaging modalities.^5^ The results showed that the gold markers were visible in all imaging modalities. Further, fiducial markers created susceptibility artifacts in T1‐weighted (T1w) and T2‐weighted (T2w) magnetic resonance (MR) images were visible as signal voids. In CT and CBCT images, the fiducial markers caused streak artifacts that were clearly observable.^5^

The visibility of the fiducial markers on MRI is particularly crucial when an MRI‐only workflow is employed, and thereby, no CT images are available for marker identification. The benefit of the MRI‐only workflow is the elimination of registration uncertainties that would otherwise arise when aligning CT and MR images.^7^, ^8^ However, distinguishing fiducial markers from calcification using only MRI images can be challenging. Multi‐echo gradient echo (MEGRE) imaging amplifies magnetic susceptibility effects with prolonged echo times, improving the visibility of markers and their differentiation from calcifications.^8^, ^9^

There is an increasing interest in a focal boost or simultaneous integrated boost to intraprostatic lesions to minimize local recurrences and maximize tumor control.^10^, ^11^ To delineate these intraprostatic lesions, diffusion‐weighted MRI (DWI) and apparent diffusion coefficient (ADC) may be used. The ADC map is also useful as an indicator of tumor aggressiveness.^12^ Studies have found that the ADC values correlate with changes before and after radiation treatment of prostate cancer.^13^, ^14^ This could indicate that the ADC values may be used for monitoring and as an indicator for follow‐up in prostate cancer treatment. Both these applications underline the need to know the geometric extension of the induced artifacts by fiducial markers in the prostate.

The standard acquisition technique for DWI is echo planar imaging (EPI). Although EPI is fast and provides great image contrast, it is sensitive to local magnetic field variations induced by, for example, fiducial markers.^15^ The low bandwidth in the phase‐encoding (PE) direction makes EPI particularly prone to distortion artifacts. Because DWI relies on EPI, these distortion artifacts and signal loss become more pronounced in DWI compared to T1w or T2w imaging.^15^ Moreover, the artifacts created by the fiducial markers also scale with the magnetic field strength.^16^, ^17^

Additionally, motion during diffusion will affect the detected signal, as phase shifts can occur that mimic or obscure diffusion effects.^18^ Therefore, there is a need to investigate the effects of fiducial markers on DWI, as they could be detrimental for target delineation and applications for tumor tissue characterization.

The artifact extension from gold fiducial markers on diffusion weighted images on a 1.5T and a 3T scanner has been investigated using a homogeneous phantom.^19^ It was concluded that the selection of PE direction and the orientation of the fiducial markers have a large impact on the artifact size in diffusion‐weighted images. Since artifacts in DWI influence the pixel values used to calculate ADC maps, the presence of fiducial markers can also alter the corresponding regions in the ADC maps.^19^

While it is recognized that fiducial markers introduce artifacts in DWI and ADC maps, there is a limited number of studies that have assessed the distance of geometrical artifact extension–especially on 3T systems, or how the visibility of the markers in ADC maps is affected by motion and varying signal‐to‐noise ratio (SNR). This gap is problematic, as there is a potential risk of obscuring small intraprostatic boost volumes in DWI or ADC maps, where the geometric accuracy is crucial, or misinterpreting ADC values when used for treatment monitoring.

The aim of this study was to determine the distances of artifact extensions caused by the intraprostatic fiducial markers in DWI and ADC maps. To achieve this, we conducted experiments using homogeneous and heterogeneous phantoms with fiducial markers inserted. Diffusion MRI was performed with the phantom in both static and moving conditions, using acquisitions with both full and reduced SNR. Further, ADC maps of the phantoms containing motion were computed. The extensions of artifacts caused by fiducial markers in PE and frequency‐encoding (FE) directions were investigated. The artifact extensions in patient DWI were also studied, and the results were compared to phantom measurements.

## 2 METHODS AND MATERIALS

In this study, a 3T GE Architect scanner was used (Software SIGNA_LX1.MR30.0_R01_2242.a, General Electric Healthcare, Milwaukee, WI, USA). Diffusion‐weighted images were acquired on homogeneous and heterogeneous phantoms using an EPI sequence (Figure S1). We used *b*‐values 0, 200, and 800 s/mm^2^ in three orthogonal directions with a TE/TR of 69/4596 ms, which is the protocol used for prostate cancer patients in our clinic. For the calculation of the ADC maps and all other image processing and evaluation, the software Hero (Hero Imaging, Umeå, Sweden) was used.

### 2.1 Homogeneous phantom

**FIGURE 1:**
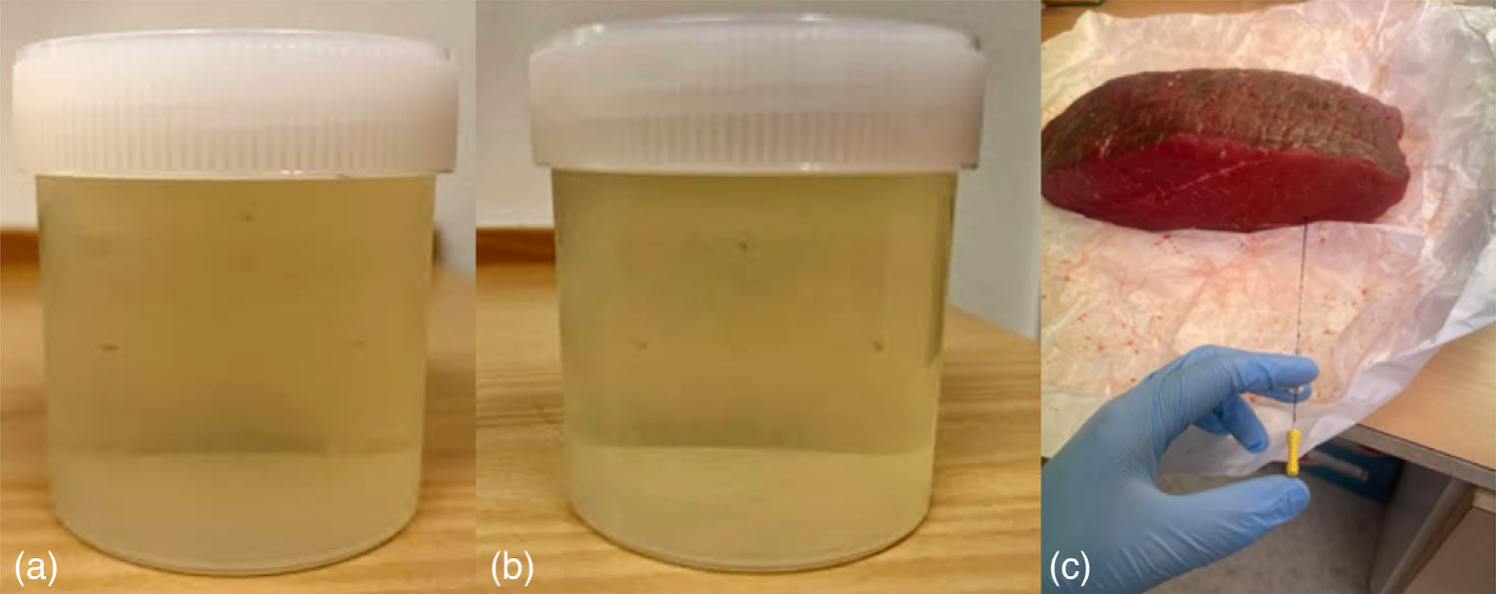
(a) Gel phantom containing three GA. (b) Gel phantom containing three GFM. (c) Sirloin with three GFM and three GA. The phantoms were scanned axially. The orientation of the GFM was chosen to simulate their position in the prostate, with the long axis placed in parallel to the axial plane.

Two nickel‐nitrate gel phantoms were created using 8 g of porcine skin powder (type A, Sigma–Aldrich) per 100 mL of water to mimic prostatic tissue, with T1‐ and T2‐relaxation times of 1200 and 550 ms, respectively. Three cylindrical gold fiducial markers (GFM) produced in‐house with a diameter of 1 mm and a length of 5 mm were inserted into one phantom, and three spherical gold anchor (GA) markers (99.5% gold and 0.5% iron; GA200‐10, Naslund Medical AB, Huddinge, Sweden), with a diameter of 1 mm, were inserted into the other phantom (Figure 1).

**FIGURE 2:**
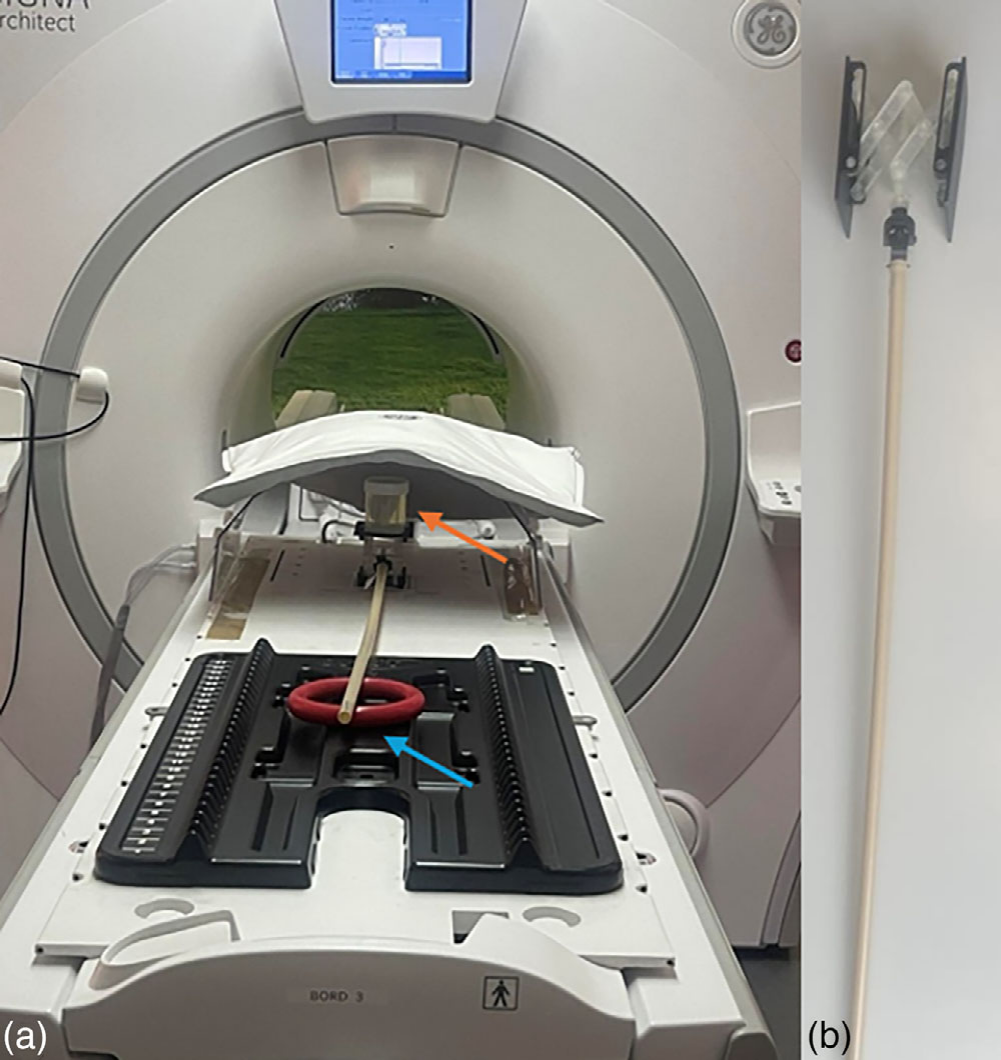
(a) Gel phantom with GFM (orange arrow). The stick was rotated to move the phantom (blue arrow). The posterior array coil, together with the anterior array coil, was utilized. (b) Close‐up view of the scissor lift.

The phantoms were imaged according to Table 1. To simulate prostate movement, the phantom with GFM was also scanned on a scissor lift that raises the platform unevenly, intentionally causing a tilt during elevation (Figure 2). This introduces simultaneous lifting in A/P, lateral translation in R/L, and rotation. The phantom was moved 1 mm (A/P) and 1 mm (R/L), 2 mm (A/P) and 2 mm (R/L), 3 mm (A/P) and 3 mm (R/L), and 4 mm (A/P) and 3 mm (R/L). Controlled motion was applied to the phantom with 30 s intervals during the acquisition using a control rod attached to the lift. To measure the magnitude of the motion, T1w images were acquired before and after DWI. This motion was also replicated in Hero, where the corresponding *b*‐value images from the first scan were displaced according to the known motion parameters. An ADC map was then generated based on the *b*‐values with the simulated motion. Moreover, ADC maps were generated using artificial motion simulation of 2 and 4 mm applied to *b* = 800 s/mm^2^ images using Hero.

**TABLE 1:** Acquisition parameters for DWI.

Parameter	DWI
Sequence	2D SE‐EPI^*^
TE [ms]	69
TR [ms]	4596
Slice thickness [mm]	3
Acquisition matrix	160×80
Number of averages	5
Acquired in plane resolution [mm]	1.5×1.5
Reconstructed in plane resolution [mm]	0.94×0.94
Bandwidth [kHz]	250
Reconstructed matrix	256×128
Air Recon DL level	High
Frequency encoding direction	R/L
Acquisition time [min]	6.31
Freq. FOV^**^	24.0
Phase FOV^***^	0.50
Number of diffusion directions	3

* SE‐EPI, Spin‐echo Echo planar imaging.

** Freq. FOV, Frequency field‐of‐view.

*** Phase FOV, Phase field‐of‐view.

Full protocol can be found in Figure S1.

DWI was also acquired on the phantom by varying the SNR. The signal is proportional to the sine of the flip angle, which is proportional to the transmitter gain;^20^ thus following relation holds between the signal and the transmit gain:

(1) $$S=S_{0}\sin\left(\frac{TG}{TG_{0}}\frac{\pi }{2}\right)$$

where *S* is the signal, *TG* is the transmitter gain, *TG_0_* is the 90° transmit gain, determined by the pre‐scan, and *S_0_* is the signal at full 90° excitation. Imaging was performed with an SNR (or *S/S_0_*) of 100%, 90%, 80%, 70%, 60%, 50%, and 40%.

The effect of lower SNR combined with motion was investigated by repeating scans with the same motion pattern for an SNR of 100%, 90%, 80%, 70%, and 60%.

### 2.2 Heterogeneous phantom

Three GFM and three GA were inserted into a 3 kg piece of sirloin measuring 33 cm in length, 20 cm in width, and 6 cm in depth, serving as a heterogeneous phantom mimicking soft tissue (Figure 1).

The heterogeneous phantom was too heavy to accomplish a precise motion pattern with the scissor lift. Therefore, a different system with plastic plates was used. Initially, images were acquired with the phantom in a baseline position, on top of two plastic plates with a height of 2 mm each. Two subsequent images with increasing shift in position were acquired by removing each plate and moving the phantom 2 mm along the left‐right, using markings on the couch.

Two ADC maps, including a reposition of 2 and 4 mm, both along PE and FE direction, respectively, were generated by combining the unique *b*‐value images from the baseline scan and the repositioned scans. Moreover, as previously mentioned, ADC maps were generated using artificial motion simulation of 2 and 4 mm applied to *b* = 800 s/mm^2^ images using Hero. Furthermore, ADC maps with lower SNR were simulated in Hero by reducing the number of averages (NEX) per *b*‐value image compared to those used in patient images (Figure S2).

### 2.3 Patient images

Ethical approval was granted by the Regional Ethical Review Board in Lund under protocols Dnr 2013/742 and Dnr 2016/801.

The artifact extension in patient images, with GFM, was assessed using line profiles. Images from eight patients who had undergone DWI with the same protocol as the phantoms were studied. Imaging parameters are provided in Table 1.

To assess the artifact extension caused by the fiducial markers in the *b*‐value images and the ADC maps, line profiles were drawn across each marker, both along PE and FE direction (Figure S3). The artifact extension was determined by visual inspection of the line profile, with the extent defined as the portion of the profile where the signal intensity deviated from the background. For each image, the mean artifact extension was calculated based on three individual marker profiles.

A linear model was fitted to artifact extension as a function of NEX and SNR, respectively. To assess whether the relationships were statistically significant, *t*‐tests on the model coefficients were used. A significance level of 5% was applied. The assumption of normality of residuals was evaluated through visual inspection of Q‐Q plots.

## 3 RESULTS

**FIGURE 3:**
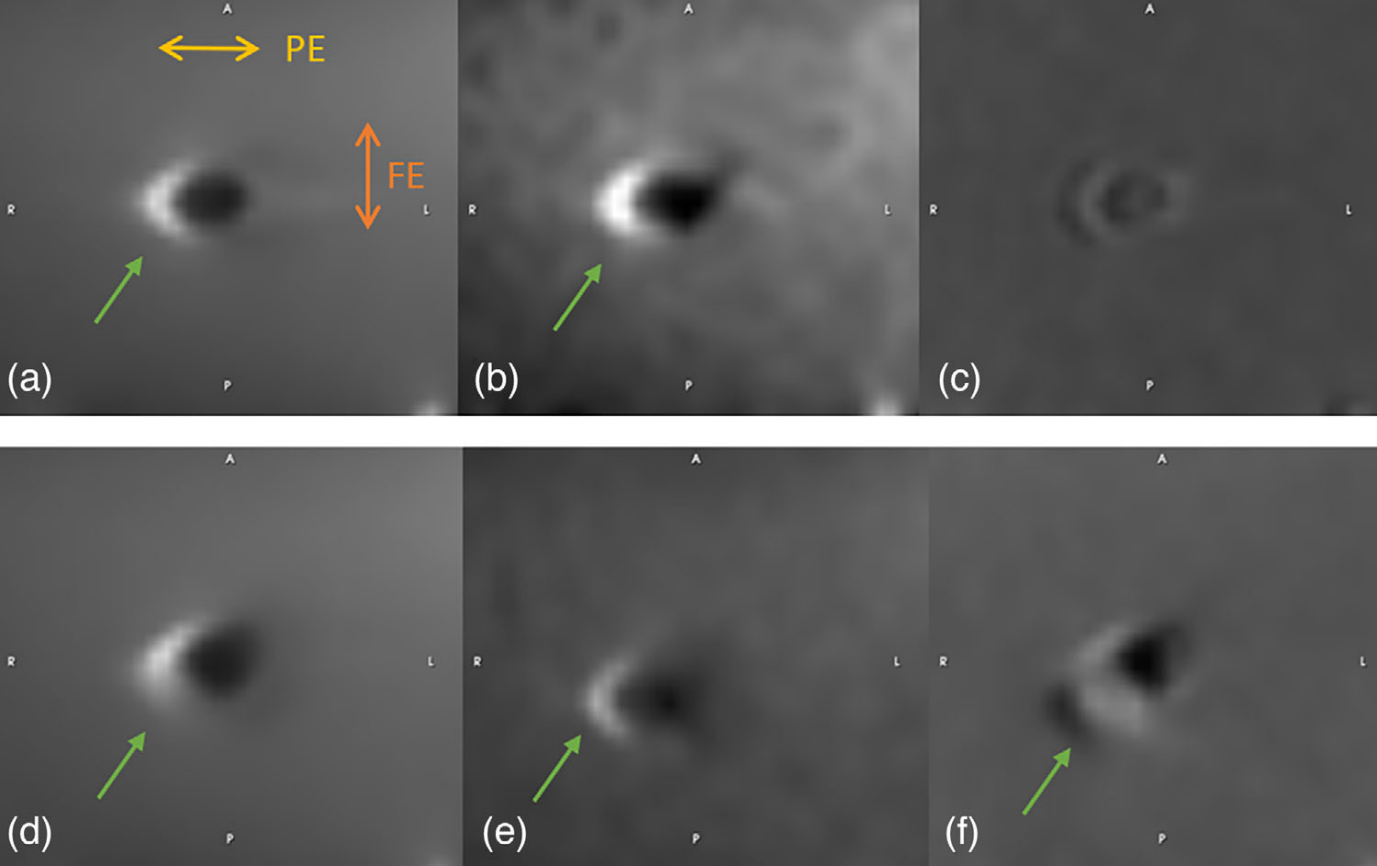
(a, b) DWI with arrows pointing out the bright pile‐up artifacts caused by GFM in homogeneous phantom images. (a) *b* = 0 s/mm^2^. (b) *b* = 800 s/mm^2^. (c) Corresponding ADC map for (a) and (b); note the absence of the bright pile‐up artifact. (d, e) repositioned unique *b*‐value images; (e) shows repositioned image relative to (d). The arrows point out the bright pile‐up artifacts caused by GFM. (d) *b* = 0 s/mm^2^. (e) *b* = 800 s/mm^2^. (f) Corresponding ADC map for (d) and (e); note the presence of a bright pile‐up artifact in (f) due to motion in DWI, which is absent in (c).

Bright pile‐up artifacts were found in the homogeneous phantom in diffusion‐weighted images along the PE direction (Figure 3). This bright region was not present in the ADC map generated from DWI, where all *b*‐value images were acquired at the same position, despite being clearly visible in the individual *b*‐value images (Figure 3a–c). In contrast, bright pile‐up artifacts were visible in the ADC map when it was generated using *b*‐value images with motion present (Figure 3d–f).

**FIGURE 4:**
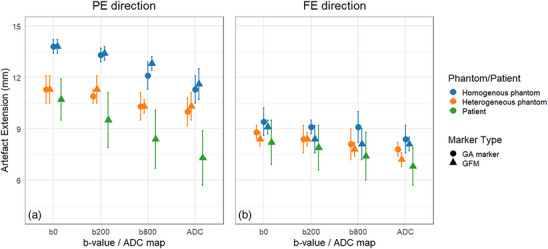
Point plot showing the mean artifact extension (± 1 SD) across different *b*‐value images and corresponding ADC maps in the PE (a) and FE (b) directions. Data are shown for three media: homogeneous (blue) and heterogeneous (orange) phantoms, and patient (green). Circles indicate artifact extensions induced by GA, and triangles indicate artifact extensions induced by GFM. The largest artifact extensions were seen in homogeneous phantom images. Smaller artifact extensions were seen in the ADC maps compared to individual *b*‐value images.

**FIGURE 5:**
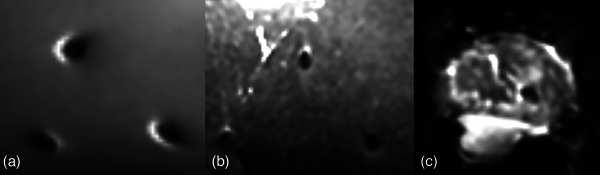
GFM in DWI (*b* = 0 s/mm^2^). (a) homogeneous phantom image. (b) heterogeneous phantom image. (c) patient image, showing the prostate. Images not to scale.

The mean artifact extensions caused by GFM and GA markers in homogeneous and heterogeneous phantoms, as well as patient images, are shown in Figure 4. The distances of artifact extensions decreased with increasing *b*‐values. Smaller artifact extensions were observed in the ADC maps compared to individual *b*‐value images. The largest artifact extensions were found along the PE direction. Larger artifact extensions were observed in homogeneous phantom images compared to patient images. The artifact extensions in homogeneous phantom images were as follows: 13.8  ±  0.4 mm / 9.1  ±  0.4 mm (PE/FE) in DWI with *b* = 0 s/mm^2^ and 11.6  ±  0.9 mm / 8.1  ±  0.4 mm (PE/FE) in the ADC map. The artifact extensions in patient images were as follows: 10.7  ±  1.2 mm / 8.2  ±  1.3 mm (PE/FE) in DWI with *b* = 0 s/mm^2^ and 7.3  ±  1.6 mm / 6.8  ±  1.1 mm (PE/FE) in the ADC map. Overall, the artifact extensions found in the heterogeneous phantom images were closer to those found in patient images compared to homogeneous phantom images (Figure 5). Similar results were found using GA markers and GFM.

**FIGURE 6:**
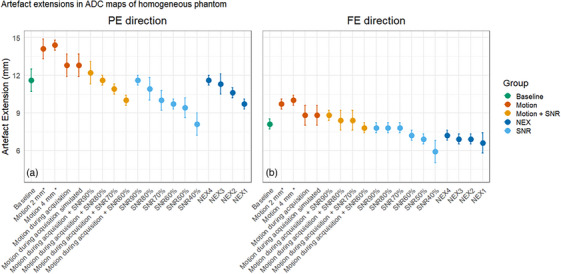
Point plot showing the mean artifact extension (± 1 SD) for different ADC maps of the homogeneous phantom in the PE (a) and FE (b) directions. Artifact extension caused by the GFM was measured under five main conditions: (1) static phantom (no motion) with full SNR (NEX = 5 per *b*‐value), referred to as baseline, (2) motion‐induced variations with full SNR, (3) motion‐induced variations in combination with varying SNR, (4) static phantom with varying NEX, and (5) static phantom with varying SNR. Motion conditions include artificial motion simulation of 2 and 4 mm applied to *b* = 800 s/mm^2^ images using Hero (*). Motion during acquisition was obtained by rotating the stick and the same motion was also simulated in Hero. ADC maps reconstructed using 1 to 4 NEX per *b*‐value image are also included, as well as those obtained under different SNR conditions by adjusting the gain. The acquisition with SNR variation was additionally repeated with induced motion by rotating the stick (Motion + SNR). Motion caused larger artifact extensions compared to the baseline. The artifact extension reduced significantly with reducing SNR (*p* < 0.001) and NEX (*p* = 0.021), respectively. No difference was obtained between artifact extensions when motion was simulated versus induced.

**FIGURE 7:**
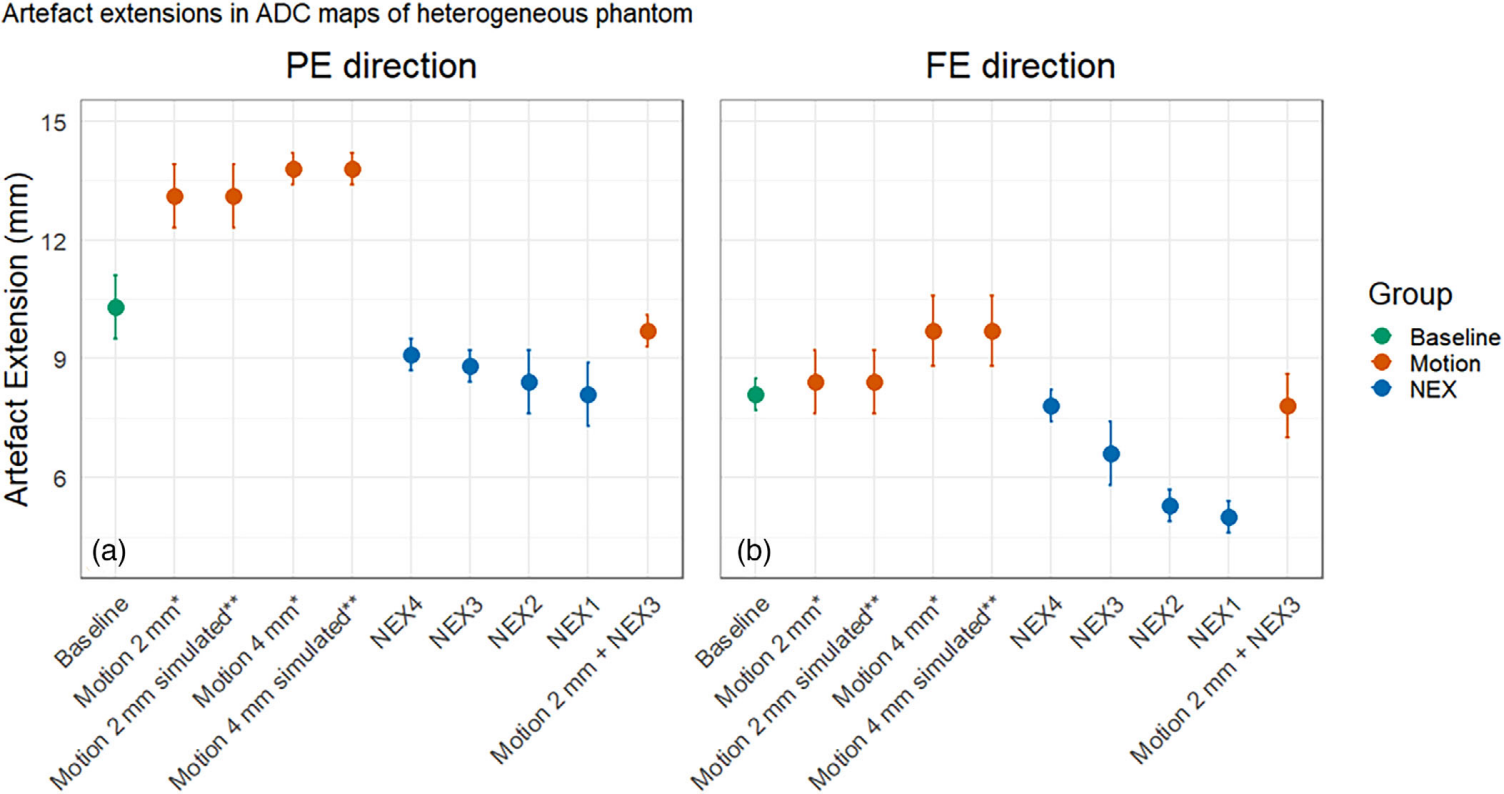
Point plot showing the mean artifact extension (± 1 SD) for different ADC maps of the heterogeneous phantom in the PE (a) and FE (b) directions. Artifact extension caused by the GFM was measured under three main conditions: (1) static phantom (no motion between *b*‐value images) with full SNR (NEX = 5 per *b*‐value), referred to as baseline, (2) motion‐induced variations with full SNR, and (3) static phantom with varying NEX. Motion conditions include manual repositioning of the phantom by 2 and 4 mm between *b*‐value acquisitions (*), and artificial motion simulation of 2 and 4 mm applied to *b* = 800 s/mm^2^ images using Hero (**). ADC maps reconstructed using 1 to 4 NEX per *b*‐value image are also included, as well as a combined motion and NEX condition (2 mm motion with 3 NEX). Motion caused larger artifact extensions compared to baseline. The artifact extension significantly reduced with reducing NEX (*p* = 0.014). No difference was obtained between artifact extensions when motion was simulated versus induced.

**FIGURE 8:**
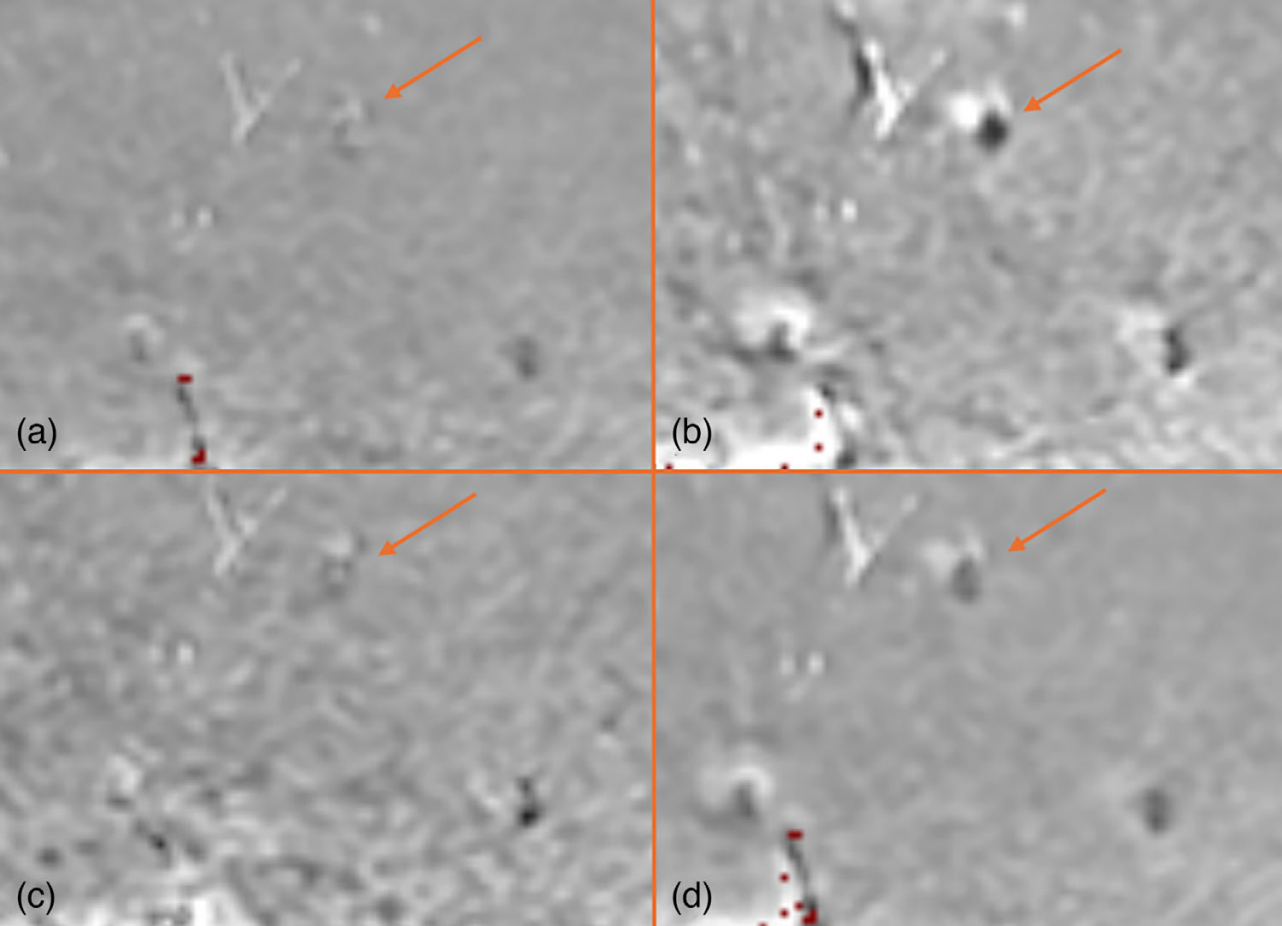
ADC maps of the heterogeneous phantom with GFM. (a) ADC map generated with the phantom in baseline position. (b) ADC map generated with a motion of 2 mm between *b* = 0 s/mm^2^ and *b* = 800 s/mm^2^. (c) ADC map generated by reducing the number of averages per *b*‐value compared to those used in patient images (3 NEX/ *b*‐value). (d) ADC map generated using 3 NEX/*b*‐value and induced motion of 2mm. Notice the reduced artifact extension with lower SNR and increased artifact extension in the presence of motion.

Motion caused larger artifact extensions compared to no motion (Figures 6, 7). A motion of 2 mm increased the artifact from 11.6 ± 0.9 mm / 8.1 ± 0.4 mm (PE/FE) to 14.1 ± 0.8 mm / 9.7 ± 0.4 mm (PE/FE) in homogeneous phantom images. In heterogeneous phantom images, the artifact extension increased from 10.3 ± 0.8 mm / 8.1 ± 0.4 mm (PE/FE) to 13.1 ± 0.8 mm / 8.4 ± 0.8 mm (PE/FE) with a motion of 2 mm (Figure 8). Additionally, there was no difference in the results for manually induced motion and simulated motion, respectively.

Artifact extension decreased along both the FE and PE directions in the homogeneous phantom images (Figure 5) as SNR decreased. A drop of 50% in SNR (through modified gain using Equation (1)) resulted in decreased artefact extension by 2.2 mm (PE) and 1.2 mm (FE). Statistical analysis using SNR as the independent variable showed a significant relationship with artifact extension (FE: *p* = 0.005, *R* ^2^ = 0.79; PE: *p* < 0.001, *R* ^2^ = 0.94). Moreover, a decrease in NEX, analyzed separately, also resulted in smaller artifact extension, which was significant (FE: *p* = 0.035, *R* ^2^ = 0.87; PE: *p* = 0.021, *R* ^2^ = 0.94). Similar trends were observed when motion was combined with reduced SNR (FE: *p* = 0.024, *R* ^2^ = 0.81; PE: *p* < 0.001, *R* ^2^ = 0.99).

For the heterogeneous phantom, a statistically significant relationship was also found between decreased NEX and decreased artifact extension along the PE direction (*p* = 0.014, *R* ^2^ = 0.87) and along the FE direction (*p* = 0.004, *R* ^2^ = 0.94).

**FIGURE 9:**
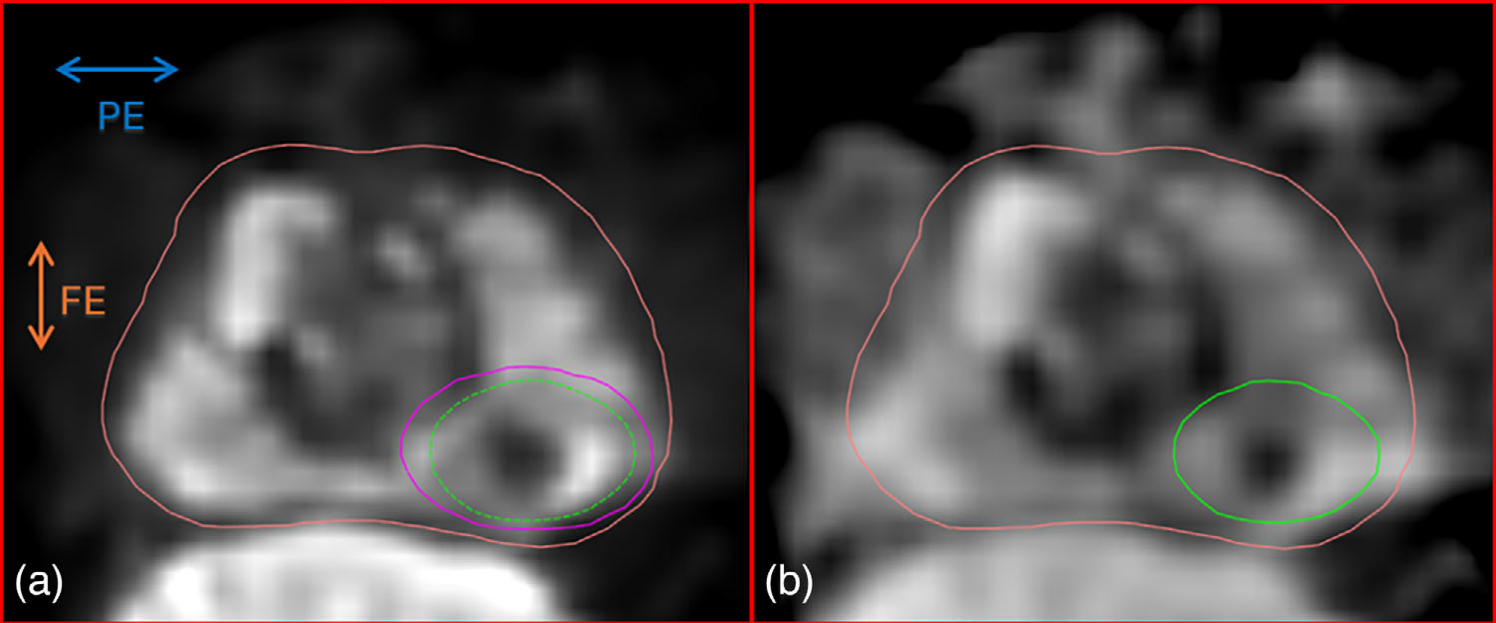
The measured artifact extensions around a GFM on an in‐vivo prostate, delineated by the outer pink ROI. (a) Diffusion weighted image (*b* = 0 s/mm^2^). The magenta ROI represents the artifact extension in DWI, while the dashed green ROI indicates the artifact extension of the corresponding ADC map superimposed on DWI. (b) ADC map. The solid green ROI delineates the artifact extension in the ADC map. Note the difference in size between the artifact extension in (a) and (b). All images were acquired using the same parameters as listed in Table 1.

The mean artifact extensions found for GFM in homogeneous phantom images are visualized on in‐vivo prostate images (Figure 9).

## 4 DISCUSSION

In this study, we investigated artifact extensions caused by fiducial markers in DWI and ADC maps. The largest artifact extensions were observed in homogeneous phantom images, smaller extensions in heterogeneous phantom images, and the smallest extensions in patient images. Motion during imaging resulted in increased artifact extension distances, whereas lower SNR reduced artifact visibility. Artifact extensions were larger in the PE direction compared to the FE direction. Overall, ADC maps showed smaller artifact extensions than diffusion‐weighted images.

The artifact extensions observed in this study were larger along the PE direction compared to the FE direction, consistent with the findings of Rylander et al. (2011), who investigated a homogeneous phantom. The mean artifact size along the PE direction, reported by Rylander et al., was 1.3 cm in diffusion‐weighted images, for 3 T, which falls within the range of the results found by this study, despite differences in acquisition protocol. During EPI acquisition, a high readout gradient is used, giving a high bandwidth in the FE direction. Consequently, spatial distortions are more pronounced in the PE direction due to the lower effective PE bandwidth.^15^ Previous studies have also investigated the artifacts from fiducial markers in MRI.^5^, ^6^ However, these focused on anatomical imaging and did not assess artifacts under motion and varying SNR conditions. Therefore, there are no directly comparable results for our findings.

In addition, the artifact extensions increased in the presence of motion. EPI‐based diffusion‐weighted imaging is particularly sensitive to local field variations, especially along the PE direction, and motion exacerbates these variations,^21^ accounting for the more pronounced variation in artifact size observed between mobile and static conditions. When motion was applied to the homogeneous phantom, artifact extension increased; however, when SNR was sufficiently reduced, the artifact size decreased significantly. These findings suggest that although motion contributes to increased artifact size, its effect may be partially masked or diminished under conditions of low SNR.

In the heterogeneous phantom, an increased extent of artifact manifestation was also observed in the presence of motion. The motion in the heterogeneous phantom was introduced either through repositioning between scans or artificial motion in Hero, whereas in the homogeneous phantom, motion was also manually induced during image acquisition. The methods of artificial simulation and repositioning in between scans were chosen for the heterogeneous phantom since it was difficult to induce a reproducible motion during acquisition. Importantly, the results in Figures 6, 7 show no difference in artifact extension between simulated motion and induced motion, indicating that the simulated motion adequately replicates the artifact effects of induced motion.

The artifact extensions found for the GA were similar to the GFM: 13.8 ± 0.4 mm versus 13.8 ± 0.4 mm along the PE direction, and 9.1 ± 0.4 mm versus 9.4 ± 0.8 mm along the FE direction, in the homogeneous phantom images. The outstanding visibility of the GA markers in MR images is attributed to their iron content. However, this will also induce artifacts.^22^ Nevertheless, in our case, no notable differences were observed in artifact extensions between the two types of markers, indicating that the small amount of iron in GA markers did not have a measurable impact on artefact size.

Further, the largest artifact extensions were observed in homogeneous phantom images. These images had the highest SNR and contrast‐to‐noise ratio (CNR), which resulted in more visible artifacts from signal voids and distortions caused by the fiducial markers. While it is reasonable to suspect that the presence of nickel in the phantom could contribute to susceptibility artifacts, control tests using gels with and without nickel showed no significant difference in artifact extension.

In contrast, the heterogeneous phantom and patient images exhibit lower CNR and SNR, which may obscure artifacts or complicate the delineation of their extent, thereby causing the artifacts to appear reduced in size. This effect was also evident in images with reduced SNR, for example, when comparing low and high SNR for homogeneous phantom images with motion. Moreover, the artifact extension gradually decreased for higher *b*‐values, which may be explained by the lower SNR in the images for higher *b*‐values. The voxels affected by artifact‐induced signal voids become harder to distinguish from the background noise in these images.

In DWI, magnetic field variations due to the difference in magnetic susceptibility between the fiducial markers and surrounding tissues caused bright pile‐up artifacts, leading to signal loss, image distortion, and a decrease in overall image quality. The overall artifact extension was lower in the ADC maps compared to diffusion‐weighted images. The ADC represents the slope of the logarithmic signal intensity as a function of *b*‐values. In homogeneous phantom images, the intensity shifts caused by the artifacts were consistent across different *b*‐values, reducing the impact on the calculated ADC values and resulting in less pronounced artifacts in the ADC maps (Figure S4). However, when motion was present in the DWI, the consistency across different b‐values was disrupted, causing a visible bright pile‐up artifact also in the corresponding ADC‐map.

In a clinical scenario, it is typical to position three fiducial markers, aligning their long axes approximately parallel to the main magnetic field and orthogonal to both the PE and FE direction. The transverse plane of a 2D acquired image, with slice‐encoding direction along superior‐inferior, is used for target delineation. Accordingly, the inclusion of radial margins around the markers is required to avoid detrimental distortions, which were observed to be in the order of ∼1 cm. Given the dimensions of the prostate and the observed artifact extensions in this study, accurate contouring of intraprostatic boost areas on DWI may be complicated in the vicinity of fiducial markers. In a clinical context, the margin distances needed due to image artifacts from one or more markers could potentially include the intended target volumes for the boost. Consequently, thoughtful consideration of the interventional procedure of marker placement becomes essential, particularly avoiding placement in or adjacent to target areas within the prostate when employing DWI or ADC images for delineation. Furthermore, ADC values within these margins could potentially be unreliable if used for monitoring treatment response.

In this study, we employed two types of markers. However, a range of different fiducial markers is commercially available, including liquid markers. Liquid markers have gained popularity due to their enhanced visibility in MRI and CT images and their lower tendency to create image artifacts compared to metal markers. However, liquid markers can introduce a chemical shift artifact in the ADC map ^23^, which may reduce their visibility on diffusion‐weighted imaging and limit the number of usable voxels for quantitative analyses. Similar to the approach used in this study, analyses may be required to assess the extent of artifacts caused by liquid markers. Other MRI sequence strategies to suppress susceptibility artifacts could also be explored; however, since our work specifically addresses artifacts in DWI, we have not focused on alternative MRI sequence strategies, which fall beyond the scope of this study.

An alternative to fiducial markers is the recently proposed method for markerless MRI‐only radiotherapy for prostate cancer treatment. It eliminates patient discomfort and artifacts related to makers, relying on MR and CBCT soft tissue matching, which were found to provide comparable accuracy to CT‐CBCT matching, with only minor differences.^24^ However, the implementation may necessitate experienced therapeutic radiographers for precise soft tissue matching. Another limitation of markerless methods may be the challenges for triggered imaging, as the latter technique relies on markers for accuracy in dose delivery.^24^

One limitation of this study is that it focuses exclusively on two types of markers. The artifact extension is also expected to vary with MRI field strength and sequence parameters; however, we chose to use the 3 T SE‐EPI protocol that is clinically applied to our patients to ensure relevance. While the absolute artifact sizes reported here are specific to this protocol, the trends we observed regarding the impact of motion and SNR are expected to apply to other markers and sequence parameters as well. The methodology employed here is reproducible and can readily be applied across different clinical and research settings. Another limitation may be the small sample size (*n* = 8) used in our analysis. Nevertheless, the consistent findings across all cases support the observations, while future studies with larger cohorts could further confirm the generalizability of the ^∼^1 cm artifact range.

In conclusion, in this study, the distances of artifact extensions in homogeneous phantoms, heterogeneous phantoms, and patient images caused by intraprostatic fiducial markers in DWI and ADC maps were investigated. The study found that the distances of artifact extensions in the ADC maps were smaller compared to individual *b*‐value images. The distances of artifact extensions, both in DWI and corresponding ADC maps, were largest in the homogeneous phantom, smaller in the heterogeneous phantom, and the smallest in the patient images. In patient images, the distances of the artefact extensions were approximately 7–11 mm (along PE) and 7–8 mm (along FE). However, they reached up to ^∼^14 mm (PE) and ^∼^9 mm (FE) in homogeneous phantom images, suggesting that the true distances of artifact extensions may be partially obscured in patient images. Further, motion in images caused larger artifact extensions, and lower SNR caused smaller artifact extensions. Nevertheless, careful consideration of marker placement remains crucial to prevent the obscuration of important anatomical structures, particularly in the delineation of small boost volumes or the distortion of ADC values in quantitative analyses.

## AUTHOR CONTRIBUTIONS

*Performing experiments*: Mizgin Coskun, Lars E. Olsson, and Patrik Brynolfsson.

All authors contributed to the study design. All authors participated in the results analysis, reviewed and edited the manuscript.

## CONFLICT OF INTEREST STATEMENT

The co‐author Christian Jamtheim Gustafsson is a consultant for GE Healthcare. The co‐author Patrik Brynolfsson is a shareholder and board member of Hero Imaging AB, developer of ‘Hero’. The remaining authors have nothing to declare.

## ETHICAL APPROVAL

Ethical approval was granted by the Regional Ethical Review Board in Lund under protocols Dnr 2013/742 and Dnr 2016/801.

## Supporting information



Supporting Information
