# Supplementary material for: Distance‐based assessment of spatial artifact extension in the prostate from fiducial markers in diffusion‐weighted magnetic resonance imaging
Source: J Appl Clin Med Phys. 2025 Nov 14;26(11):e70348. doi: 10.1002/acm2.70348 (PMC12618179; doi:10.1002/acm2.70348)
Supplement: Supplementary file 1 — Supporting Information [file ACM2-26-e70348-s001.docx]

Supplementary


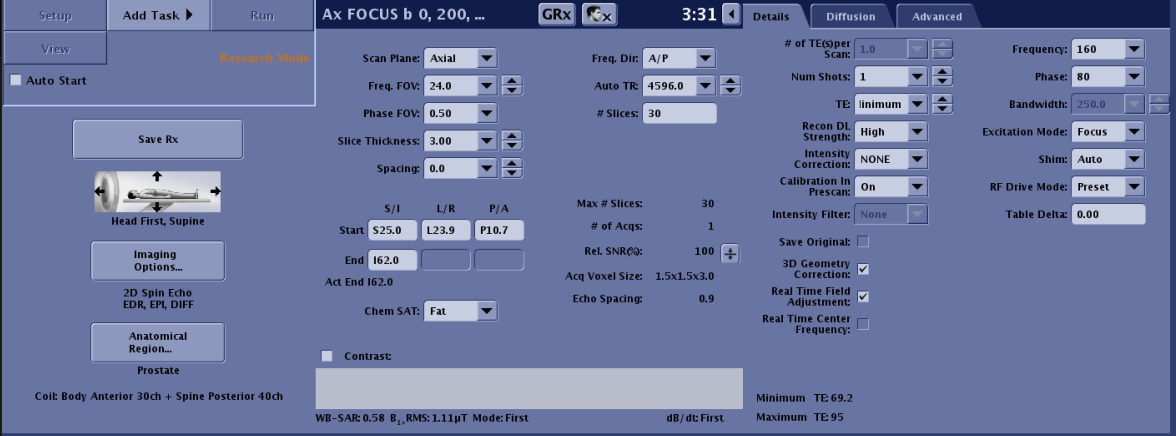

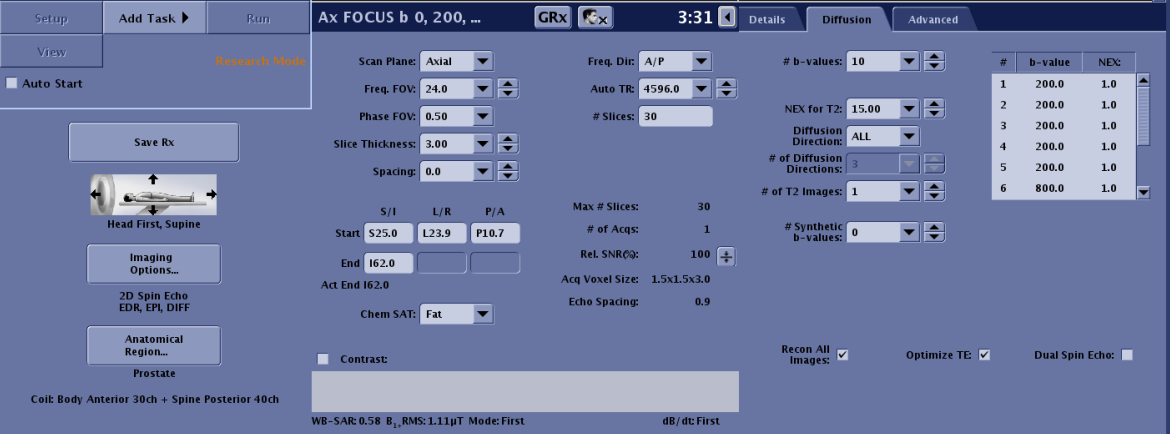

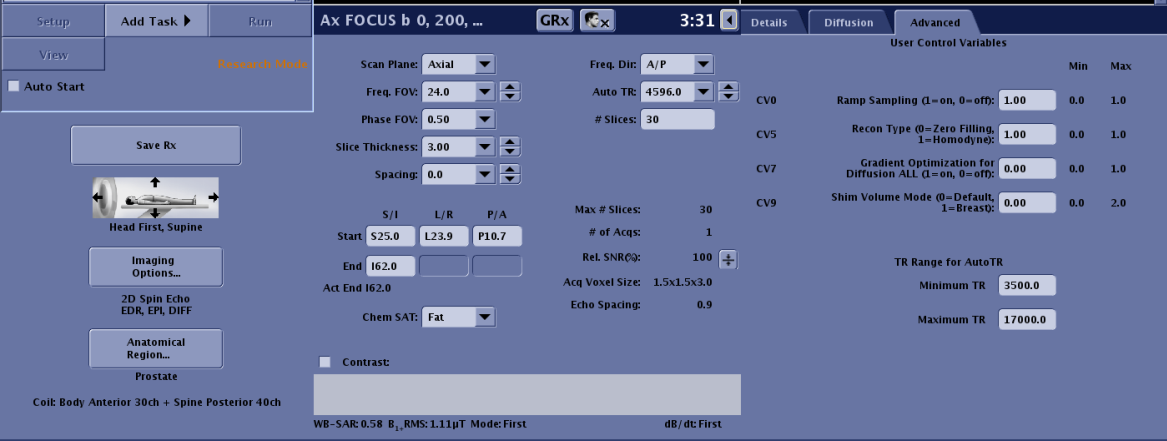


Figure S1: The diffusion-weighted MRI sequence on 3T GE Architect used in this experiment.


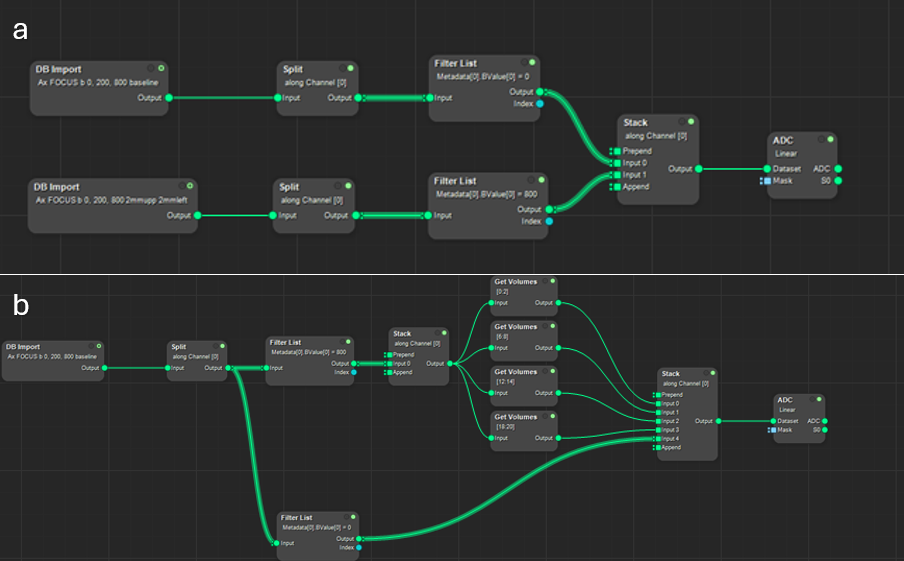


Figure S2: Workflow in Hero for generating ADC maps of the heterogeneous phantom. a) ADC map created using a 2 mm displacement between the b = 0 s/mm² and b = 800 s/mm² images. Specifically, the b = 0 s/mm² image was acquired with the phantom in its baseline position, while the b = 800 s/mm² image was from a separate acquisition where the phantom had been shifted by 2 mm. These two images were then combined to generate the ADC map. b) ADC map generated using 3 NEX (instead of 5 NEX). To do this, diffusion images with b = 800 s/mm² were filtered, and specific volumes were selected to simulate an acquisition with 3 NEX before generating the ADC map.


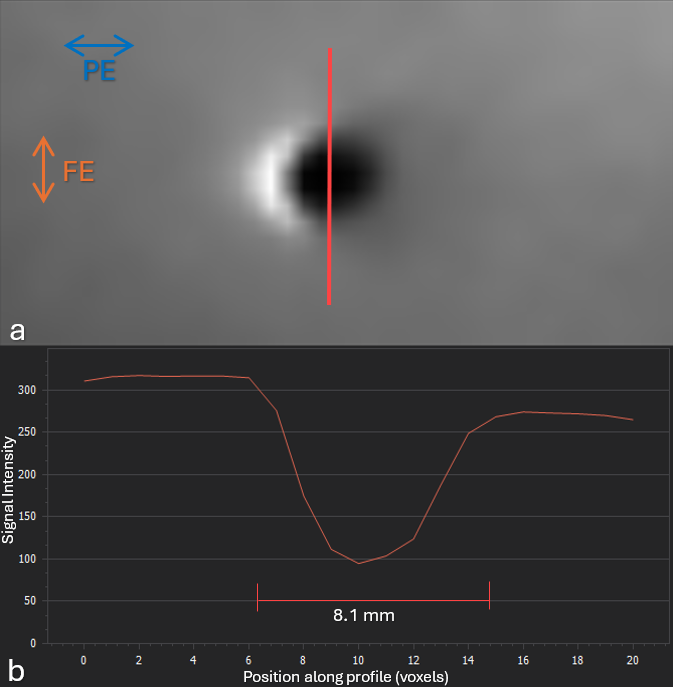


Figure S3: Artifact measurement using a line profile. a) Diffusion-weighted phantom image (b=0 s/mm²) with a fiducial marker; the red line indicates the position of the profile drawn along the FE direction. b) Corresponding signal intensity profile. Artifact extension was defined where the signal deviated from the background plateau, corresponding to the signal dip across the marker.


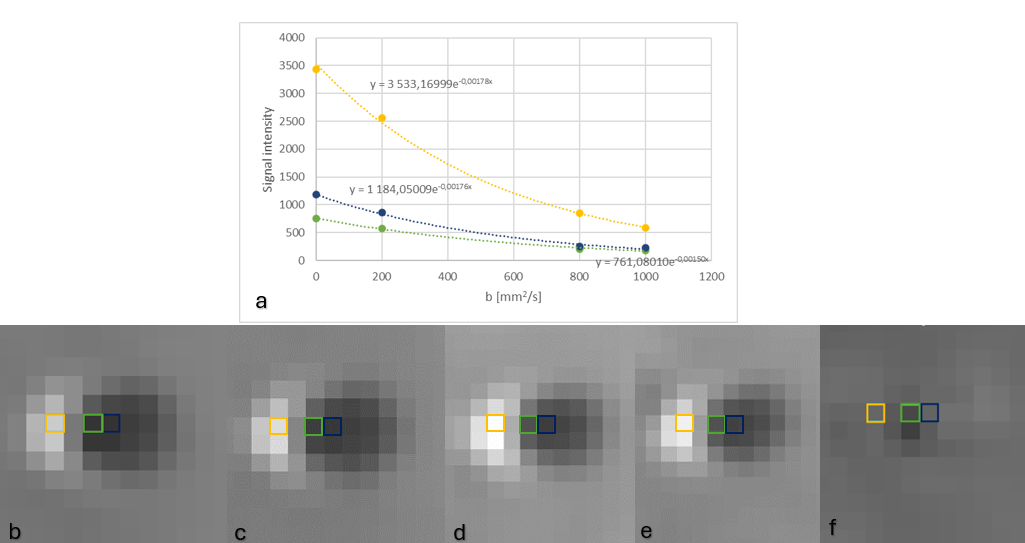


Figure S4: a) Exponential decay of signal intensity as a function of the b-values. The exponent in each equation represents the ADC value for the marked voxels in the ADC map shown in F. b-e) DWI, with b=0, 200, 800 & 1000 s/mm² (from left to right), showing a marker with bright pile-up artifact. f) Corresponding ADC map. Note that the bright voxel in DWI (yellow box) and dark voxel (blue box) have similar ADC values in a), explaining why the bright pile-up artifact is not visible in the ADC map.
